# How to Make More Published Research True

**DOI:** 10.1371/journal.pmed.1001747

**Published:** 2014-10-21

**Authors:** John P. A. Ioannidis

**Affiliations:** 1 Meta-Research Innovation Center at Stanford (METRICS), Stanford University, Stanford, California, United States of America; 2 Department of Medicine, Stanford Prevention Research Center, Stanford, California, United States of America; 3 Department of Health Research and Policy, Stanford University School of Medicine, Stanford, California, United States of America; 4 Department of Statistics, Stanford University School of Humanities and Sciences, Stanford, California, United States of America

## Abstract

In a 2005 paper that has been accessed more than a million times, John Ioannidis explained why most published research findings were false. Here he revisits the topic, this time to address how to improve matters.

*Please see later in the article for the Editors' Summary*

**Summary Points**

- Currently, many published research findings are false or exaggerated, and an estimated 85% of research resources are wasted.
- To make more published research true, practices that have improved credibility and efficiency in specific fields may be transplanted to others which would benefit from them—possibilities include the adoption of large-scale collaborative research; replication culture; registration; sharing; reproducibility practices; better statistical methods; standardization of definitions and analyses; more appropriate (usually more stringent) statistical thresholds; and improvement in study design standards, peer review, reporting and dissemination of research, and training of the scientific workforce.
- Selection of interventions to improve research practices requires rigorous examination and experimental testing whenever feasible.
- Optimal interventions need to understand and harness the motives of various stakeholders who operate in scientific research and who differ on the extent to which they are interested in promoting publishable, fundable, translatable, or profitable results.
- Modifications need to be made in the reward system for science, affecting the exchange rates for currencies (e.g., publications and grants) and purchased academic goods (e.g., promotion and other academic or administrative power) and introducing currencies that are better aligned with translatable and reproducible research.

The achievements of scientific research are amazing. Science has grown from the occupation of a few dilettanti into a vibrant global industry with more than 15,000,000 people authoring more than 25,000,000 scientific papers in 1996–2011 alone [1]. However, true and readily applicable major discoveries are far fewer. Many new proposed associations and/or effects are false or grossly exaggerated [2],[3], and translation of knowledge into useful applications is often slow and potentially inefficient [4]. Given the abundance of data, research on research (i.e., meta-research) can derive empirical estimates of the prevalence of risk factors for high false-positive rates (underpowered studies; small effect sizes; low pre-study odds; flexibility in designs, definitions, outcomes, analyses; biases and conflicts of interest; bandwagon patterns; and lack of collaboration) [3]. Currently, an estimated 85% of research resources are wasted [5].

## Effective Interventions

We need effective interventions to improve the credibility and efficiency of scientific investigation. Some risk factors for false results are immutable, like small effect sizes, but others are modifiable. We must diminish biases, conflicts of interest, and fragmentation of efforts in favor of unbiased, transparent, collaborative research with greater standardization. However, we should also consider the possibility that interventions aimed at improving scientific efficiency may cause collateral damage or themselves wastefully consume resources. To give an extreme example, one could easily eliminate all false positives simply by discarding all studies with even minimal bias, by making the research questions so bland that nobody cares about (or has a conflict with) the results, and by waiting for all scientists in each field to join forces on a single standardized protocol and analysis plan: the error rate would decrease to zero simply because no research would ever be done. Thus, whatever solutions are proposed should be pragmatic, applicable, and ideally, amenable to reliable testing of their performance.

Currently, major decisions about how research is done may too often be based on convention and inertia rather than being highly imaginative or evidence-based [5]–[15]. For example, there is evidence that grant reviewers typically have only modest CVs and most of the top influential scientists don't review grant applications and don't get funded by government funds, even in the United States [6], which arguably has the strongest scientific impact at the moment than any other country (e.g., in cumulative citations). Non-meritocratic practices, including nepotism, sexism, and unwarranted conservatism, are probably widespread [7]. Allegiance and confirmation biases are powerful in scientific processes [8],[9]. For healthcare and clinical practice, while evidence-based medicine has grown stronger over time, some argue that it is currently in crisis [10] and “evidence-based” terminology has been usurped to promote expert-based beliefs and industry agendas [11]. We have little experimental evidence on how peer review should be done and when (e.g., protocol-based, manuscript-based, post-publication) [5],[12],[13] or on how research funds should be allocated [14],[15]. Many dominant scientific structures date back to the Middle Ages (e.g., academic hierarchies) or the 17th century (e.g., professional societies, journal publishing), but their suitability for the current growth of science is uncertain. At the same time, there is an obvious tension in hoping for decisions to be both more imaginative and more evidence-based; it may be the case that the bureaucracy and practice of science require different people with different skill sets, and it may even be that a system too focused on eliminating unfair discrimination also eliminates the reasonable discrimination required to make wise choices. While we could certainly introduce changes that made science worse, we could also purposefully introduce ones to make it better.

One option is to transplant into as many scientific disciplines as possible research practices that have worked successfully when applied elsewhere. Box 1 lists a few examples that are presented in more detail here.

**Box 1. Some Research Practices that May Help Increase the Proportion of True Research Findings**

- Large-scale collaborative research
- Adoption of replication culture
- Registration (of studies, protocols, analysis codes, datasets, raw data, and results)
- Sharing (of data, protocols, materials, software, and other tools)
- Reproducibility practices
- Containment of conflicted sponsors and authors
- More appropriate statistical methods
- Standardization of definitions and analyses
- More stringent thresholds for claiming discoveries or ‘‘successes’’
- Improvement of study design standards
- Improvements in peer review, reporting, and dissemination of research
- Better training of scientific workforce in methods and statistical literacy

Adoption of large-scale collaborative research with a strong replication culture [16] has been successful in several biomedical fields: in particular, in genetic and molecular epidemiology. These techniques have helped transform genetic epidemiology from a spurious field [17] to a highly credible one [18]. Such practices could be applied to other fields of observational research and beyond [19].

Replication has different connotations for different settings and designs. For basic laboratory and preclinical studies, replication should be feasible as a default, but even in those cases, there should be an a priori understanding of the essential features that are needed to be replicated and how much heterogeneity is acceptable [20]. For some clinical research, replication is difficult, especially for very large, long-term, expensive studies. The prospect of replication needs to be considered and incorporated up front in designing the research agenda in a given field [12]. Otherwise, some questions are not addressed at all or are addressed by single studies that are never replicated, while others are subjected to multiple unnecessary replications or even redundant meta-analyses combining them [21].

Registration of randomized trials [22] (and, more recently, registration of their results [23]) has enhanced transparency in clinical trials research and has allowed probing of selective reporting biases [24],[25], even if not fully remedying them. It may show redundancy and allow better visualizing of the evolution of the total corpus of research in a given field. Registration is currently proposed for many other types of research, including both human observational studies [26] and nonhuman studies [27].

Sharing of data, protocols, materials, and software has been promoted in several -omics fields, creating a substrate for reproducible data practices [28]–[31]. Promotion of data sharing in clinical trials may similarly improve the credibility of clinical research [32]. Some disadvantages have been debated, like the potential of multiple analysts performing contradicting analyses, difficulties with de-identification of participants, and the potential for parties to introduce uncertainty for results that hurt their interests, as in the case of diesel exhaust and cancer risk [33].

Dissociation of some research types from specific conflicted sponsors or authors has been proposed (not without debate) for designs as diverse as cost-effectiveness analyses [34], meta-analyses [35],[36], and guidelines [37]. For all of these types of research, involvement of sponsors with conflicts has been shown to spin more favorable conclusions.

Adoption of more appropriate statistical methods [38], standardized definitions and analyses and more stringent thresholds for claiming discoveries or “successes” [39] may decrease false-positive rates in fields that have to-date been too lenient (like epidemiology [40], psychology [41],[42], or economics [43]). It may lead them to higher credibility, more akin to that of fields that have traditionally been more rigorous in this regard, like the physical sciences [44].

Improvements in study design standards could improve the reliability of results [45]. For example, for animal studies of interventions, this would include randomization and blinding of investigators [27]. There is increasing interest in proposing checklists for the conduct of studies to be approved [46],[47], making it vital to ensure both that checklist items are indeed essential and that claims of adherence to them are verifiable.

Reporting, review, publication, dissemination, and post-publication review of research shape its reliability. There are currently multiple efforts to improve and standardize reporting (e.g., as catalogued by the EQUATOR initiative [48]) and multiple ideas about how to change peer review (by whom, how, and when) and dissemination of information [25],[49]–[51].

Finally, proper training and continuing education of scientists in research methods and statistical literacy are also important [47].

## Stakeholders

As we design, test, and implement interventions on research practices, we need to understand who is affected by and shaping research [5],[52],[53]. Scientists are only one group in a larger network (Table 1) in which different stakeholders have different expectations. Stakeholders may cherish research for being publishable, fundable, translatable, or profitable. Their expectations are not necessarily aligned with one another. Scientists may continue publishing and getting grants without making real progress, if more publications and more grants are all that matters. If science is supported primarily by private investors who desire patents and profit, this may lead to expedited translation and discoveries that work (or seem to work) but also barriers against transparency and sharing of information. Corporate influence may subvert science for the purposes of advertising, with papers in influential journals, prestigious society meetings, and a professorate system of opinion leaders becoming branches of their marketing department [11],[54]. The geography of scientific production changes rapidly; e.g., soon there will be more English language papers from China than from Europe and the US [55]. Research efforts are embedded in wider societies, which have provided scientific developments that differ according to time period and location. What can be done to enhance the capacity of science to flourish and to assess and promote this capacity across cultures that may vary in attitudes toward skepticism, inquisitiveness, and contrarian reasoning? Different stakeholders have their own preferences about when reproducibility should be promoted or shunned. Pharmaceutical industry teams have championed reproducibility in pre-clinical research [56],[57] because they depend on pre-clinical academic investigations accurately pinpointing useful drug targets. Conversely, the industry is defensive about data sharing from clinical trials [30], which occurs at a point in the product development when re-analyses may correctly or incorrectly [58] invalidate evidence supporting drugs in which it has already invested heavily.

**Table 1:** Some major stakeholders in science and their extent of interest in research and its results from various perspectives; typical patterns are presented (exceptions do occur).

	Extent of interest in research results			
	Publishable	Fundable	Translatable	Profitable
*Scientists*	+++	+++	+	
Industry – sales and marketing				+++
Industry – R & D			+++	+++
Private investors, including hedge funds			++	+++
Public funders – open (e.g. NIH, NSF)	++		+	
Public funders – closed (e.g. military)			+++	
Not-for-profit funders/philanthropists	++		+++	
Journal editors	+++			+
For-profit publishers	+			+++
Professional and scientific societies	+			
Universities	+	+++		+
Not-for-profit research institutions	+++	+++	+	+
Supporting non-scientific staff		+++		
Hospitals and other professional facilities offering services related to science			+	+++
Other financial entities that are affected by these services (e.g. insurance)				+++
Governments and state/federal authorities				++
Consumers of products and services			+++	

Dynamics between different stakeholders are complex. Moreover, sometimes the same person may wear many stakeholder hats; e.g., an academic researcher may also be journal editor, spin-off company owner, professional society officer, government advisor, and/or beneficiary of the industry.

## Research Currencies

Publications and grants are key “currencies” in science (Table 2). They purchase academic “goods” such as promotion and other power. Academic titles and power add further to the “wealth” of their possessor. The exact exchange rate of currencies and the price of academic goods [59] may vary across institutional microenvironments, scientific disciplines and circumstances, and are also affected by each microenvironment's fairness or unfairness (e.g., nepotism, cronyism, or corruption). Administrative power, networking, and lobbying within universities, inbred professional societies, and academies further distort the picture. This status quo can easily select for those who excel at gaming the system, producing prolifically mediocre and/or irreproducible research; controlling peer review at journals and study sections; enjoying sterile bureaucracy, lobbying, and maneuvering; and promoting those who think and act in the same way.

**Table 2:** An illustration of different exchange rates for various currencies and wealth items in research.

	Different examples of reward systems		
	Current	Change 1	Change 2
**CURRENCIES**			
Publication (per unit)	Win 1	No value	No value
Replicated publication (per unit)	Win 1	Win 2	Win 2
Successfully translated publication (per unit)	Win 1	Win 5	Win 5
Refuted publication (per unit)	Win 1	Lose 1	Lose 1
Sharing data, protocols, analysis codes (per unit)	No value	Win 2	Win 2
Contribution to peer-review (per unit)	No value	Win 2	Win 2
Contribution to education/training (per unit)	No value	Win 1	Win 1
Grant funding (per one R01)	Win 5	Win 5	Lose 5
**OTHER WEALTH ITEMS**			
Assistant professor, title in good university	Win 3	Win 3	No value
Associate professor, title in good university	Win 10	Win 10	No value
Tenured professor, title in good university	Win 20	Win 20	No value
Team leader/director			
Per 1 doctoral student/post-doc	Win 2	Win 2	Lose 2
Administrative power, networking, lobbying	Win up to 200	No value	Lose up to 200

There are also opportunities in grasping the importance of the key currencies. For example, registration of clinical trials worked because all major journals adopted it as prerequisite for publication [60], a major reference currency in the reward chain. Conversely, interesting post-publication review efforts such as PubMed Commons [61] have so far not fulfilled their potential as progressive vehicles for evaluating research, probably because there is currently no reward for such post-publication peer review.

## Modifying the Reward System

The reward system may be systematically modified [62]. Modifying interventions may be anywhere from fine-tuning to disruptive. Table 2 compares the status quo (first column) against two potential modifications of the reward system, with “Change 2” being more prominent than “Change 1.”

The current system values publications, grants, academic titles, and previously accumulated power. Researchers at higher ranks have more papers and more grants. However, scholars at the very top of the ladder (e.g., university presidents) have modest, mediocre, or weak publication and citation records [63]. This might be because their lobbying dexterity compensates for their lack of such credentials, and their success comes at the expense of other worthier candidates who would bring more intellectual rigor and value to senior decision making; equally, it could be because they excel at the bureaucratic work necessary to keep the mind-boggling academic machine going, and their skills enable more scientifically gifted colleagues to concentrate on research. The current system does not reward replication—it often even penalizes people who want to rigorously replicate previous work, and it pushes investigators to claim that their work is highly novel and significant [64]. Sharing (data, protocols, analysis codes, etc.) is not incentivized or requested, with some notable exceptions [65]–[67]. With lack of supportive resources and with competition (“competitors will steal my data, my ideas, and eventually my funding”), sharing becomes even disincentivized. Other aspects of scientific citizenship, such as high-quality peer review, are not valued. Peer review can be a beneficial process, acting as a safety net and a mechanism for augmenting quality. It can also be superficial, lead to only modest improvements of the reviewed work, and allow for the acceptance of blatantly wrong papers [68],[69]. That it is so little valued and rewarded is not calculated to encourage its benefits and minimize its harms.

The currency values shown in Table 2 are for illustrative purposes, to provoke thought about the sort of rewards that bias the process of scientific work. Such currency values will vary across microenvironments and specific fields and situations. A putative currency value of 1 for a publication unit (e.g., a first- or senior-authored paper in a highly respectable journal in the field), 5 for a sizeable investigator grant (e.g., an R01 in the US), and 2 for a post-doctoral fellow means that a scientist would find equivalent value in publishing five such papers as first or senior author as in getting an R01 as a principal investigator, or in publishing two such papers as in getting a post-doctoral fellow to work for her. Moreover, what constitutes a publication unit may also vary across fields: in fields in which people publish sparingly, a single article may be enough to define a publication unit, while in fields in which it is typical for people to put their names in hundreds of papers, often with extreme multi-authorship, ten such papers may be needed for an equivalent publication unit. Inflationary trends like redundant and salami publication [70] and unwarranted multi-authorship have made the publication currency lose relative value over time in many disciplines. Adjustments for multi-authorship are readily feasible [71],[72]. Knowledge of individual contributions in each paper would allow even better allocation of credit [73].

In the first example of a proposed modification of the reward system shown in Table 2, the purchasing power of publications is primarily differentiated depending on their replication and translation status. Value is given to sound ideas and results that are replicated and reproducible [74] rather than publication per se. Further value is given to publications that lead to things that work, like effective treatments, diagnostic tests, or prognostic tools that demonstrably improve important outcomes in clinical trials. Additional value is obtained for sharing and for meaningful participation in peer review and educational activities of proven efficacy. A peer reviewer or an editor occasionally may contribute the same value as an author.

The second example of a proposed modification shown in Table 2 carries even greater changes to the reward system. Besides the changes adopted in the first example, obtaining grants, awards, or other powers are considered negatively unless one delivers more good-quality science in proportion. Resources and power are seen as opportunities, and researchers need to match their output to the opportunities that they have been offered—the more opportunities, the more the expected (replicated and, hopefully, even translated) output. Academic ranks have no value in this model and may even be eliminated: researchers simply have to maintain a non-negative balance of output versus opportunities. In this deliberately provocative scenario, investigators would be loath to obtain grants or become powerful (in the current sense), because this would be seen as a burden. The potential side effects might be to discourage ambitious grant applications and leadership.

Such trade-offs clarify that when it comes to modifying the structure of scientific careers, as when modifying pathophysiology in an attempt to fight illness, interventions can do harm as well as good. Given the complexity of the situation, interventions should have their actual impacts fairly and reliably assessed.

## Moving Forward

The extent to which the current efficiency of research practices can be improved is unknown. Given the existing huge inefficiencies, however, substantial improvements are almost certainly feasible. The fine-tuning of existing policies and more disruptive and radical interventions should be considered, but neither presence nor absence of revolutionary intent should be taken as a reliable surrogate for actual impact. There are many different scenarios for the evolution of biomedical research and scientific investigation in general, each more or less compatible with seeking truthfulness and human well-being. Interventions to change the current system should not be accepted without proper scrutiny, even when they are reasonable and well intended. Ideally, they should be evaluated experimentally. The achievements of science are amazing, yet the majority of research effort is currently wasted. Interventions to make science less wasteful and more effective could be hugely beneficial to our health, our comfort, and our grasp of truth and could help scientific research more successfully pursue its noble goals.
